# Sealing Ability of Orthograde MTA and CEM Cement in Apically Resected Roots Using Bacterial Leakage Method

**Published:** 2013-08-01

**Authors:** Saeed Moradi, Reza Disfani, Kiarash Ghazvini, Mahdi Lomee

**Affiliations:** a Department of Endodontics, Dental Materials Research Center, Mashhad University of Medical Sciences, Mashhad, Iran; b Department of Endodontics, Dental Research Center, Mashhad University of Medical Sciences, Mashhad, Iran; c Department of Microbiology, Mashhad University of Medical Sciences, Mashhad, Iran; d Department of Endodontics, Dental School, Mazandaran University of Medical Sciences, Mazandaran, Iran

**Keywords:** Apicoectomy, Bacterial Leakage, Calcium Enriched Mixture, CEM cement, Mineral Trioxide Aggregate, Retrograde Obturation, Root Canal Filling Materials, Seal

## Abstract

**Introduction:**

The aim of this in vitro study was to determine the sealing ability of orthograde ProRoot mineral trioxide aggregate (MTA) and calcium enriched mixture (CEM) cement as root-end filling materials.

**Materials and Methods:**

Fifty four extracted single-rooted human teeth were used. The samples were randomly divided into 3 experimental groups. In group A and B, 4 mm of WMTA and CEM cement were placed in an orthograde manner and 3 mm of apices were resected after 24 hours. In group C the apical 3 mm of each root was resected and the root-end prepared with ultrasonic tips to a depth of 3 mm and subsequently, then filled with MTA. The apical sealing ability was performed with bacterial leakage method. Statistical analysis was carried out with Chi-square test.

**Results:**

There were no significant differences in the extent of bacterial leakage between the three experimental groups (P>0.05).

**Conclusion:**

Based on the limitations of this in vitro study, we concluded that MTA and CEM cement can be placed in an orthograde manner when there is a potential need for root-end surgery.

## Introduction

Studies had shown that microorganisms and their by-products play an important role in the pathogenesis of pulpal and periapical diseases [1, 2]. The goal of nonsurgical endodontic therapy (initial and retreatment) includes the reduction/elimination of these irritants from the root canal system and three dimensional filling of the canal space [3-5]. In these instances, where nonsurgical root canal treatment was unsuccessful, periradicular surgery may be necessary to facilitate healing [6], Different materials have been proposed to seal the root-end cavity, including amalgam, reinforced zinc oxide-eugenol, glass ionomer cements, composite resins and more recently mineral trioxide aggregate (MTA) [7-9].

MTA has several potential clinical specifications, such as superior sealing ability [10-12], setting in moist environment [13], biocompatibility [14-16] and bacteriocidal activity [17, 18]. However, delayed setting time, poor handling characteristics and high price are the disadvantages of this biomaterial [19].

Recently, calcium enriched mixture (CEM) cement consisting of different calcium/phosphate components was developed as a water-based tooth-colored endodontic biomaterial. CEM cement has shown acceptable outcomes compared to MTA, when used as root-end filling material [20-24].

In cases where anatomic access during endodontic surgery is difficult for root-end cavity preparation and restoration, the clinician may place the root-end filling material orthogradly while nonsurgical retreatment is performed [25]. In this simple method, root-end preparation and root-end filling is not required, making the procedure more practical and efficient compared to conventional methods; however, root resection may alter the apical seal of previously set orthograde material.

The purpose of this *in vitro* study was to evaluate the sealing ability of orthograde MTA and CEM in resected roots with bacterial leakage method and compare that with retrograde MTA during conventional endodontic surgery.

## Material and Methods

### Tooth preparation

Fifty four extracted fully developed human maxillary anterior teeth with single, straight canals without cracks, caries, restoration and resorption were selected. Teeth were immersed in 2.6% sodium hypochlorite for approximately one hour to remove organic materials from the root surfaces. Any remaining tissue was carefully removed with a curette; the teeth were then stored in physiologic saline and kept moist before and during the experiment. The teeth were decoronated using a #330 bur (Komet, Lemgo, Germany) in a high-speed handpiece with water coolant to have a root length of 15 mm. Access cavity was prepared and working length determined by inserting a #15 K-file (Dentsply, Maillefer, Ballaigues, Switzerland) into the canal until it was just visible at the apical foramen, 1mm was subtracted from this value. The specimen were instrumented using step-back technique to a #40 K-file up to 3 mm shorter than working length. The coronal portions were flared using Gates-Glidden burs (Dentsply, Maillefer, Ballaigues, Switzerland). The canals were irrigated between files with 2 mL of 5.25% sodium hypochlorite. Smear layer was removed with 5 mL of 17% EDTA followed by a final flush of 5 mL of sodium hypochlorite. After instrumentation was completed, the canals were dried with sterile paper point (Ariadent, Tehran, Iran).

### Obturating the canals

Teeth were randomly divided into 3 experimental groups of 15 specimens and 2 control groups (one sample in positive control group and two samples in negative control).

In experimental group A and B, tooth-colored MTA (ProRoot; Tulsa Dental, Tulsa, OK, USA) and CEM (Bionique Dent, Tehran, Iran) mixed with liquid according to the manufacturer’s recommendation and coronally introduced into the canal from an orthograde direction with a messing gun (EndoGun; Medidenta, Woodside, NY, USA) up to 3 mm shorter than working length. The materials were initially condensed with the thick end of moistened paper points and subsequently compacted with endodontic pluggers (Hu-Friedy, Chicago, IL, USA) to create a 4 mm thick apical plug in 3 mm of working length. Digital radiographs were taken to ensure void-free MTA/CEM placement and plug thickness. The remaining canal space was left unfilled, but a cotton pellet moistened with saline was placed in contact with MTA and CEM. The coronal access was sealed with cavit (ESPE, Norristown, PA, USA) and the samples were stored in a humidor at 37°C and 95% humidity for 48 hours. After material had set, the 4 mm root-end of samples in both groups was resected at 90 degree to the long axis of the tooth, to expose the previously set material. At the end, there was a 4 mm apical plug of both materials in the apical part of the resected roots.

In experimental group C, the apical 3 mm of each sample was resected at 90 degree to the long axis of the tooth and root-end preparation was done to a depth of 3 mm using ultrasonic tips in an ultrasonic unit (Spartan, Fenton, MO, USA). To provide an intracanal matrix to condense the root-end filling material against, a gutta-percha cone (Ariadent, Tehran, Iran) was adapted to the apical part of the canal, leaving a root-end void of 3 mm confirmed by periodontal probe and radiographs. Canals and the cavities were dried with sterile paper points before placing the root-end fillings. MTA was mixed, placed in to the root-end cavity with a messing gun and condensed with endodontic pluggers. After the cavity was completely filled, the intracanal barrier was replaced with moistened paper points, the density and depth of the filling material was verified by the radiographs, and then the teeth were temporary sealed. This group served as a gold standard.

Three samples that were instrumented but not obturated were used as positive control group, demonstrating bacterial leakage through the entire length of the canal. In negative control groups, the canals were obturated according to each experimental group.

Two layers of nail varnish were applied to the surfaces of all teeth in the experimental and positive control groups, excluding the resected apical portion and access cavity to prevent bacterial microleakage through the lateral canals or other discontinuities in the cementum. In negative control groups, the entire root was covered with two layers of nail varnish except the access cavity.

### Bacterial microleakage

A dual-chamber anaerobic bacteria model was modified from a technique developed by Torabinejad *et al.* [26]. The upper chamber of the leakage apparatus was assembled by cutting 5mm off the end of an Eppendorf tube (Sigma-Aldrich Co., Hamburg, Germany), and then the samples were inserted into the tubes until the roots protruded through the end. The junction between each tube and root was sealed with cyanoacrylate and sticky wax to prevent leakage. The assembled apparatus was gas sterilized using ethylene oxide for an 8 hour cycle using the Anprolene AN 74C Gas sterilizer (Andersen Products Inc., Haw River, NC, USA). A volume of 8-10 mL sterile Muller-Hinton Broth (Merck, Darmstadt, Germany) was introduced in to sterile 13×100 mm disposable culture tubes (Pouyan Teb Co., Tehran, Iran), as a lower chamber of the leakage apparatus. The previously assembled portions of the tooth (upper chamber) were then inserted in the culture tubes under aseptic condition, so that a minimum 2-3 mm of the apical part of the each root was immersed in Muller-Hinton Broth. The junction between the Eppendorf and culture tubes was sealed with Parafilm tapes (Parafilm M, Laboratory Film; American National Can, Chicago, IL, USA). To ensure sterilization, the whole system was incubated at 37°C for 3 days. Any test showing signs of turbidity in Muller- Hinton Broth was discarded. Two millimeters of Muller-Hinton Broth was inoculated with 9×10^8^ CFU/mL (Mc Farland no.3) of *Enterococus (E.) faecalis* (ATCC 29212) to form a bacterial suspension that was added to the upper chamber every 2 days.The culture tubes were observed every day for turbidity of the broth in the lower chamber, which indicated bacterial growth. The day of turbidity was recorded and the experiment was conducted for 90 days. To confirm the purity of *E. faecalis* in the Muller-Hinton Broth, a sample was taken from the culture tube and cultivated.

Statistical analysis of the data was accomplished using Chi-squared test. A probability value of less than 0.05 was considered to be significant.

## Results

**Figure 1.**
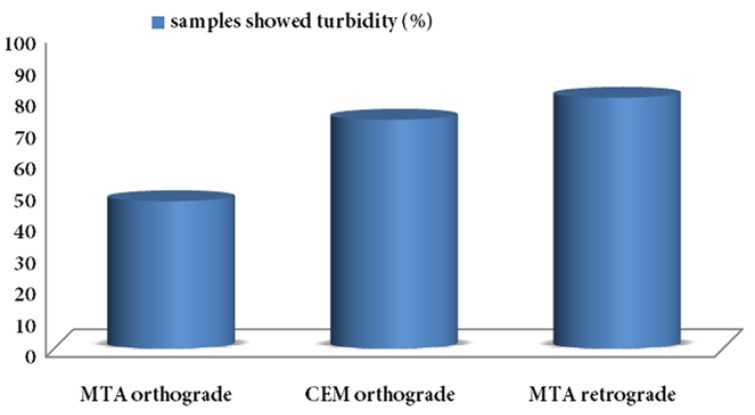
Samples showed broth turbidity 90 days after canal filling with MTA and CEM

At the end of 90 days, 7 samples in group A (46.7%), 11 samples in group B (73.3%) and 12 samples in group C (80%) showed broth turbidity (Figure 1).

The positive control groups, which were not filled with the materials showed bacterial leakage within 24 hours. The specimens in the negative control groups did not show any turbidity at 90 days (end of experiment).

**Figure 2.**
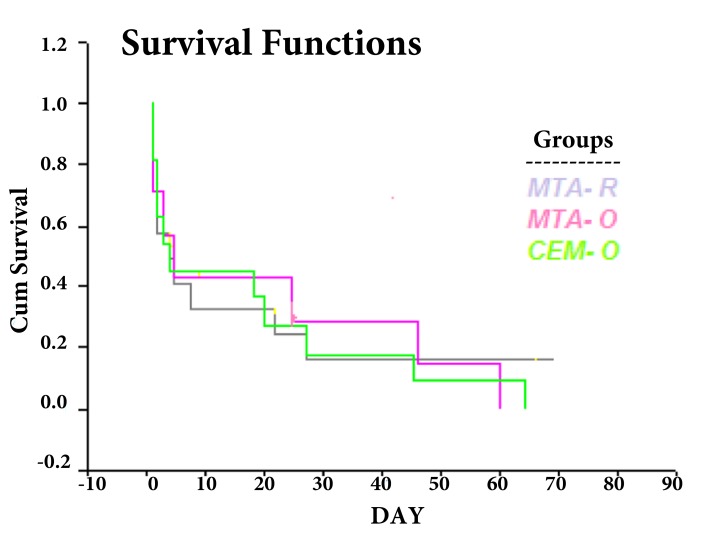
Data distributions of survival test in experimental groups

In group A, 2 samples in the first day and 5 samples after 3, 5, 25, 46 and 60 days of the experiment showed turbidity of the broth. In group B, 2 samples in the first and second days and 7 samples after 3, 4, 18, 20, 27, 45 and 64 days of the experiment showed bacterial microleakage. In group C, 2 samples in the first day, 3 samples in the second day, 5 samples after 4, 5, 8, 22, 27 days and 2 samples after 69 day of the experiment showed turbidity in the lower chamber. There were no significant differences for bacterial leakage between three experimental groups (*P*>0.05). Survival test showed distributions of survival rate are the same in all experimental groups (Figure 2).

## Discussion

The results of this *in vitro* experimental study suggest that apical sealing ability of orthograde MTA and CEM plugs after root-end resection did not differ from the conventional MTA retrofillings. MTA and CEM obturants were used alone in the root and the rest of the canal was left unfilled. This enables us to measure accurately the absolute microleakage of MTA and CEM without interfering with the sealing capacity of gutta-percha and sealer. This technique has been used by other researchers [3, 27-29].

Four-millimeter thickness of apical plug was assigned, that was in accordance with Hachmeister *et al.* study [30]. Hong *et al.* [29] and de Leimburg *et al.* [31] also used 2 mm and 1, 2 and 3 mm thickness plugs that all had an acceptable seal.

Bacterial leakage method was used to assess microleakage; according to Timpawat *et al.* [32]. This method is considered to be of greater clinical and biological relevance than the dye penetration method. *E. faecalis* is frequently used, because it is a part of the normal mouth flora and is commonly found in infection with aerobic and anaerobic bacteria.

In the present study, 4 samples of MTA orthograde (group A) showed bacterial microleakage during the first five days of the experiment; however, only 3 samples leaked in the rest of the study. Also, 6 samples of CEM orthograde (group B) showed turbidity of the broth at this period of time and just 5 samples had microleakage continually over the 90 days. It is clear that half of the leaked samples of both groups occurred in the first five days of the study. Montellano* et al.* studied the apical sealing ability of MTA with bacteria microleakage method over 30 days and reported similar results [3]. Approximately all of the samples had microleakage during the first 3 days, which was matched with the results of our study.

Kim *et al.* reported that 8 samples out of 11 of MTA orthograde group of the study showed microleakage after 60 days. Their results concur well with our study [28].

Our results differ from those of Al-Kahtani *et al.* who reported no leakage during 70 days in 5 mm MTA orthograde apical plug [33]. The differences in results could be attributed to increased thickness of the obturating material and dissimilar type of bacteria. They utilized *Actinomyces viscosis* used for leakage assessment.

The results of our study disagreed with Al-Hezaimi *et al. *which was conceivable [34]. They obturated the entire length of the canal with white and grey MTA and showed that only one sample in grey MTA group and 4 samples in white MTA group (out of 11) had microleakage after 42 days. Increased thickness of MTA resulted in better resistance against bacterial leakage, similar to Al-Kahtani *et al.* study [33].

Hachmeister *et al.* did not show any difference between 1 and 4 mm MTA orthograde apical plug in immature teeth, nearly all had leakage after 70 days compared with 20% leakage of MTA root-end fillings used as controls [30]. They concluded that, the intracanal delivery technique is contributes to the leakage, not the MTA. During retrograde placement, MTA is well condensed in to the root-end preparation against a matrix such as gutta-percha and the adaptation is directly visualized. In immature teeth, orthograde delivery is technique sensitive and condensation is limited due to the lack of the apical matrix. In our study, MTA and CEM orthograde plugs were placed in teeth with block canals and subsequently the root end was resected. In this case we flared coronal portion of the canal to increase access to apical part and place the material in an appropriate point and condense the material as well.

Lamb *et al.* evaluated apical sealing ability of different thickness of MTA and concluded that root resection did not affect the sealing ability of MTA when at least 3 mm of the material remained [35]. Andelin *et al.* also reported that the resection of set MTA did not alter its sealing ability [25]. In an *in vivo* study, Habibi *et al.* declared that there were no significant differences between healing process in hard tissue formation in set and fresh MTA during surgery on cat teeth [36]. We can therefore confidently surmise that root resection did not affect the sealing ability of set MTA and CEM.

## Conclusion

Based on the results of this *in vitro* experiment, MTA and CEM biomaterials can be placed in an orthograde manner when there is a potential need for endodontic surgery.

## References

[1] Kakehashi S, Stanley HR, Fitzgerald RJ (1965). The Effects of Surgical Exposures of Dental Pulps in Germ-Free and Conventional Laboratory Rats.. Oral Surg Oral Med Oral Pathol..

[2] Moller AJ, Fabricius L, Dahlen G, Ohman AE, Heyden G (1981). Influence on periapical tissues of indigenous oral bacteria and necrotic pulp tissue in monkeys.. Scand J Dent Res..

[3] Montellano AM, Schwartz SA, Beeson TJ (2006). Contamination of tooth-colored mineral trioxide aggregate used as a root-end filling material: a bacterial leakage study.. J Endod..

[4] Bidar M, Moradi S, Forghani M, Bidad S, Azghadi M, Rezvani S, Khoynezhad S (2010). Microscopic evaluation of cleaning efficiency of three different nickel-titanium rotary instruments.. Iran Endod J..

[5] Talati A, Moradi S, Forghani M, Monajemzadeh A (2013). Shaping ability of nickel-titanium rotary instruments in curved root canals.. Iran Endod J..

[6] Yildirim T, Er K, Taşdemir T, Tahan E, Buruk K, Serper A (2010). Effect of smear layer and root-end cavity thickness on apical sealing ability of MTA as a root-end filling material: a bacterial leakage study.. Oral Surg Oral Med Oral Pathol Oral Radiol Endod..

[7] Torabinejad M, Watson TF, Pitt Ford TR (1993). Sealing ability of a mineral trioxide aggregate when used as a root end filling material.. J Endod..

[8] Chong BS, Pitt Ford TR, Watson TF, Wilson RF (1995). Sealing ability of potential retrograde root filling materials.. Endod Dent Traumatol..

[9] Kim S, Kratchman S (2006). Modern endodontic surgery concepts and practice: a review.. J Endod..

[10] Lee SJ, Monsef M, Torabinejad M (1993). Sealing ability of a mineral trioxide aggregate for repair of lateral root perforations.. J Endod..

[11] Oraie E, Ghassemi AR, Eliasifar G, Sadeghi M, Shahravan A (2012). Apical sealing ability of MTA in different liquid to powder ratios and packing methods.. Iran Endod J..

[12] Yavari HR, Samiei M, Shahi S, Aghazadeh M, Jafari F, Abdolrahimi M, Asgary S (2012). Microleakage comparison of four dental materials as intra-orifice barriers in endodontically treated teeth.. Iran Endod J..

[13] Torabinejad M, Higa RK, McKendry DJ, Pitt Ford TR (1994). Dye leakage of four root end filling materials: effects of blood contamination.. J Endod..

[14] Yildirim T, Gencoglu N, Firat I, Perk C, Guzel O (2005). Histologic study of furcation perforations treated with MTA or Super EBA in dogs' teeth.. Oral Surg Oral Med Oral Pathol Oral Radiol Endod..

[15] Abbasipour F, Akheshteh V, Rastqar A, Khalilkhani H, Asgari S, Janahmadi M (2012). Comparing the effects of mineral trioxide aggregate and calcium enriched mixture on neuronal cells using an electrophysiological approach.. Iran Endod J..

[16] Mozayeni MA, Salem Milani A, Marvasti LA, Mashadi Abbas F, Modaresi SJ (2009). Cytotoxicity of cold ceramic compared with MTA and IRM.. Iran Endod J..

[17] Torabinejad M, Hong CU, Ford TR, Kettering JD (1995). Antibacterial effects of some root end filling materials.. J Endod..

[18] Mohammadi Z, Shalavi S (2011). Effect of hydroxyapatite and Bovine serum Albumin on the antibacterial activity of MTA.. Iran Endod J..

[19] Camilleri J, Montesin FE, Di Silvio L, Pitt Ford TR (2005). The chemical constitution and biocompatibility of accelerated Portland cement for endodontic use.. Int Endod J..

[20] Asgary S, Shahabi S, Jafarzadeh T, Amini S, Kheirieh S (2008). The properties of a new endodontic material.. J Endod..

[21] Asgary S, Eghbal MJ, Parirokh M (2008). Sealing ability of a novel endodontic cement as a root‐end filling material.. J Biomed Mater Res A..

[22] Milani AS, Shakouie S, Borna Z, Sighari Deljavan A, Asghari Jafarabadi M, Pournaghi Azar F (2012). Evaluating the effect of resection on the sealing ability of MTA and CEM cement.. Iran Endod J..

[23] Asgary S, Hasheminia M, Nejad SL (2010). Sealing ability of MTA and CEM cement as root-end fillings of human teeth in dry, saliva or blood-contaminated conditions.. Iran Endod J..

[24] Kazem M, Eghbal MJ, Asgary S (2010). Comparison of bacterial and dye microleakage of different root-end filling materials.. Iran Endod J..

[25] Andelin WE, Browning DF, Hsu GH, Roland DD, Torabinejad M (2002). Microleakage of resected MTA.. J Endod..

[26] Torabinejad M, Ung B, Kettering JD (1990). In vitro bacterial penetration of coronally unsealed endodontically treated teeth.. J Endod..

[27] Yildirim T, Taşdemir T, Orucoglu H (2009). The evaluation of the influence of using MTA in teeth with post indication on the apical sealing ability.. Oral Surg Oral Med Oral Pathol Oral Radiol Endod..

[28] Kim US, Shin SJ, Chang SW, Yoo HM, Oh TS, Park DS (2009). In vitro evaluation of bacterial leakage resistance of an ultrasonically placed mineral trioxide aggregate orthograde apical plug in teeth with wide open apexes: a preliminary study.. Oral Surg Oral Med Oral Pathol Oral Radiol Endod..

[29] Hong ST, Bae KS, Baek SH, Kum KY, Lee W (2008). Microleakage of accelerated mineral trioxide aggregate and Portland cement in an in vitro apexification model.. J Endod..

[30] Hachmeister DR, Schindler WG, Walker WA, 3rd, Thomas DD (2002). The sealing ability and retention characteristics of mineral trioxide aggregate in a model of apexification.. J Endod..

[31] de Leimburg ML, Angeretti A, Ceruti P, Lendini M, Pasqualini D, Berutti E (2004). MTA obturation of pulpless teeth with open apices: bacterial leakage as detected by polymerase chain reaction assay.. J Endod..

[32] Timpawat S, Amornchat C, Trisuwan WR (2001). Bacterial coronal leakage after obturation with three root canal sealers.. J Endod..

[33] Al-Kahtani A, Shostad S, Schifferle R, Bhambhani S (2005). In-vitro evaluation of microleakage of an orthograde apical plug of mineral trioxide aggregate in permanent teeth with simulated immature apices.. J Endod..

[34] Al-Hezaimi K, Naghshbandi J, Oglesby S, Simon JH, Rotstein I (2005). Human saliva penetration of root canals obturated with two types of mineral trioxide aggregate cements.. J Endod..

[35] Lamb EL, Loushine RJ, Weller RN, Kimbrough WF, Pashley DH (2003). Effect of root resection on the apical sealing ability of mineral trioxide aggregate.. Oral Surg Oral Med Oral Pathol Oral Radiol Endod..

[36] Habibi M, Ghoddusi J, Habibi A, Mohtasham N (2011). Healing Process Following Application of Set or Fresh Mineral Trioxide Aggregate as a Root-End Filling Material.. Eur J Dent..

